# Prediction of visual impairment in retinitis pigmentosa using deep learning and multimodal fundus images

**DOI:** 10.1136/bjo-2021-320897

**Published:** 2022-07-27

**Authors:** Tin Yan Alvin Liu, Carlthan Ling, Leo Hahn, Craig K Jones, Camiel JF Boon, Mandeep S Singh

**Affiliations:** 1 Wilmer Eye Institute, Johns Hopkins Hospital, Baltimore, Maryland, USA; 2 Department of Ophthalmology, University of Maryland Medical System, Baltimore, Maryland, USA; 3 Department of Ophthalmology, Amsterdam UMC Locatie AMC, Amsterdam, The Netherlands; 4 Malone Center for Engineering in Healthcare, Whiting School of Engineering, Johns Hopkins University, Baltimore, Maryland, USA; 5 Department of Ophthalmology, Leiden University Medical Center, Leiden, The Netherlands; 6 Department of Genetic Medicine, School of Medicine, Johns Hopkins University, Baltimore, Maryland, USA

**Keywords:** degeneration, diagnostic tests/investigation, dystrophy, macula, retina

## Abstract

**Background:**

The efficiency of clinical trials for retinitis pigmentosa (RP) treatment is limited by the screening burden and lack of reliable surrogate markers for functional end points. Automated methods to determine visual acuity (VA) may help address these challenges. We aimed to determine if VA could be estimated using confocal scanning laser ophthalmoscopy (cSLO) imaging and deep learning (DL).

**Methods:**

Snellen corrected VA and cSLO imaging were obtained retrospectively. The Johns Hopkins University (JHU) dataset was used for 10-fold cross-validations and internal testing. The Amsterdam University Medical Centers (AUMC) dataset was used for external independent testing. Both datasets had the same exclusion criteria: visually significant media opacities and images not centred on the central macula. The JHU dataset included patients with RP with and without molecular confirmation. The AUMC dataset only included molecularly confirmed patients with RP. Using transfer learning, three versions of the ResNet-152 neural network were trained: infrared (IR), optical coherence tomography (OCT) and combined image (CI).

**Results:**

In internal testing (JHU dataset, 2569 images, 462 eyes, 231 patients), the area under the curve (AUC) for the binary classification task of distinguishing between Snellen VA 20/40 or better and worse than Snellen VA 20/40 was 0.83, 0.87 and 0.85 for IR, OCT and CI, respectively. In external testing (AUMC dataset, 349 images, 166 eyes, 83 patients), the AUC was 0.78, 0.87 and 0.85 for IR, OCT and CI, respectively.

**Conclusions:**

Our algorithm showed robust performance in predicting visual impairment in patients with RP, thus providing proof-of-concept for predicting structure-function correlation based solely on cSLO imaging in patients with RP.

**WHAT IS ALREADY KNOWN ON THIS TOPIC**

- The efficient conduct of adequately powered clinical trials in retinitis pigmentosa (RP) is hampered by the need to screen relatively large numbers of patients to find those who fit the inclusion criteria.

**WHAT THIS STUDY ADDS**

- Structure-function correlation based solely on confocal scanning laser ophthalmoscopy imaging in patients with RP can be predicted using deep learning (DL).

**HOW THIS STUDY MIGHT AFFECT RESEARCH, PRACTICE OR POLICY**

- DL-based estimation of visual acuity using optical coherence tomography images may enable efficient screening of potential subjects in future RP research studies or clinical trials.

## Introduction

Retinitis pigmentosa (RP) is the most prevalent group of inherited retinal dystrophy (IRD) in the world, with an estimated incidence of 1 in 4000 persons.^1^ In recent years, significant advancement has been made in the field of IRD with the Food and Drug Administration approval of voretigene neparvovec (Luxturna) for the treatment of *RPE65*-mediated IRD.^2^ According to www.clinicaltrials.gov (accessed 16 May 2021), there are 39 active interventional clinical trials for RP that are currently recruiting or enrolling subjects, and an additional 10 active studies that are not yet recruiting. Specific gene therapy targets for RP include *MERTK, PDE6A, PDE6B, RPGR* and *MYO7A* gene mutations, among others.^3 4^ Mutation-agnostic modalities being developed include cell therapy (clinicalTrials.gov identifier: NCT04604899, NCT02464436) and antioxidant therapy (NCT03063021) approaches.

The efficient conduct of adequately powered clinical trials in RP is hampered by the need to screen relatively large numbers of patients to find those that fit the inclusion criteria. Typically, inclusion criteria include visual field (VF) and visual acuity (VA) parameters. These parameters are potentially susceptible to patient-dependent and/or operator-dependent variability,^5 6^ and are labour-intensive and time-consuming to measure. The problem of variability is increased by the fact that patients are typically dispersed across many different hospitals and countries. In response to this problem, surrogate biomarkers that are based on confocal scanning laser ophthalmoscopy (cSLO) imaging have been studied and deployed. cSLO modalities include optical coherence tomography (OCT), short-wavelength fundus autofluorescence (SW-FAF) and infrared (IR) reflectance. Screening of potential RP trial subjects based on cSLO may offer the advantages of being time-efficient, objective and relatively operator-independent. The ellipsoid zone (EZ) line length^7^ on OCT has been shown to correlate with the edges of the VF in patients with RP. The foveal EZ width is currently accepted as a structural surrogate biomarker of VF size. It has gained popularity as a parameter for subject selection and also as a clinical trial outcome measure, supported by data from the EZ Working Group that validated the robust structure-function correlation of this parameter with VF indices.^8^ Another imaging surrogate biomarker of VF with potential utility in clinical trials is the size of the hyperautofluorescent ring on SW-FAF imaging.^9^

In contrast to VF, selecting a single cSLO-based surrogate biomarker for VA appears to be more challenging because multiple structural parameters appear to correlate with VA. In diabetic macular oedema, multivariate analysis has shown that central subfield thickness (CST), signal intensity and photoreceptor outer segment thickness correlate with VA.^10^ Studies in other disease contexts have shown relationships between VA and EZ integrity,^11^ external limiting membrane (ELM),^12^ outer retinal hyper-reflective foci^13^ and cone outer segments tips (COST) line integrity,^14^ among others. This complexity underscores the challenge of selecting a single OCT parameter as a surrogate biomarker for VA in RP because epiretinal membrane, outer retinal hyper-reflective foci, increased CST due to cystoid macular oedema (CME) and disruptions in ELM, COST and EZ frequently co-exist in RP.

Our goal was to further understand structure-function correlation of cSLO parameters with VA in RP. Specifically, we aimed to determine the feasibility of developing a cSLO-based model to predict visual impairment in RP using deep learning (DL). We chose a VA cut-off of Snellen 20/40, as evidence suggests that significant impairment in activities of daily living (ADL) occurs when the vision in the better-seeing eye is <20/40.^15–18^ We chose to use DL as the machine learning technique of choice as DL is particularly adept at pattern recognition. The feasibility of using DL for this purpose was supported by the recent work of Kawczynski *et al*, in which DL techniques were used to predict VA from OCT data in neovascular age-related macular degeneration.^19^ Briefly, DL processes are representation learning methods that use multilayered neural networks, the parameters of which are iteratively updated by backpropagating gradients with respect to the desired output.^20^ DL has been used to classify images, often on par with human experts, across different ophthalmology diseases, such as age-related macular degeneration (AMD), diabetic retinopathy and glaucoma.^21–26^ In this study, we chose to use cSLO imaging because it is widely available and can reliably be repeated and tracked over time. Furthermore, we hypothesised that combining two modalities (OCT and IR) would enhance the performance of the cSLO-based prediction model over using OCT alone. To enhance the rigour of our work, we leveraged distinct datasets from the USA (Johns Hopkins University (JHU)) and Europe (Amsterdam University Medical Centers (AUMC)). This approach ensured the separation of subjects for training and testing.

## Materials and methods

### Datasets

The JHU dataset included patients with a clinical diagnosis of RP. Inclusion criteria: phenotypic findings consistent with RP that included bone spicule pigmentation in the midperipheral retina on biomicroscopy, loss of the EZ in the peripheral macula on OCT, constriction of the Goldmann visual field test and typical full field electroretinogram (ERG) findings of rod-cone dysfunction consistent with RP. Exclusion criteria: visually significant media opacities and images not centred on the central macula. The JHU dataset was used for 10-fold cross-validations and internal testing. The AUMC dataset was used for independent, external testing of the trained models. All patients included in the AUMC dataset had disease-causing variants as confirmed by genetic testing. Both eyes of each patient were included, and the data were partitioned on a patient level.

**Figure 1:**
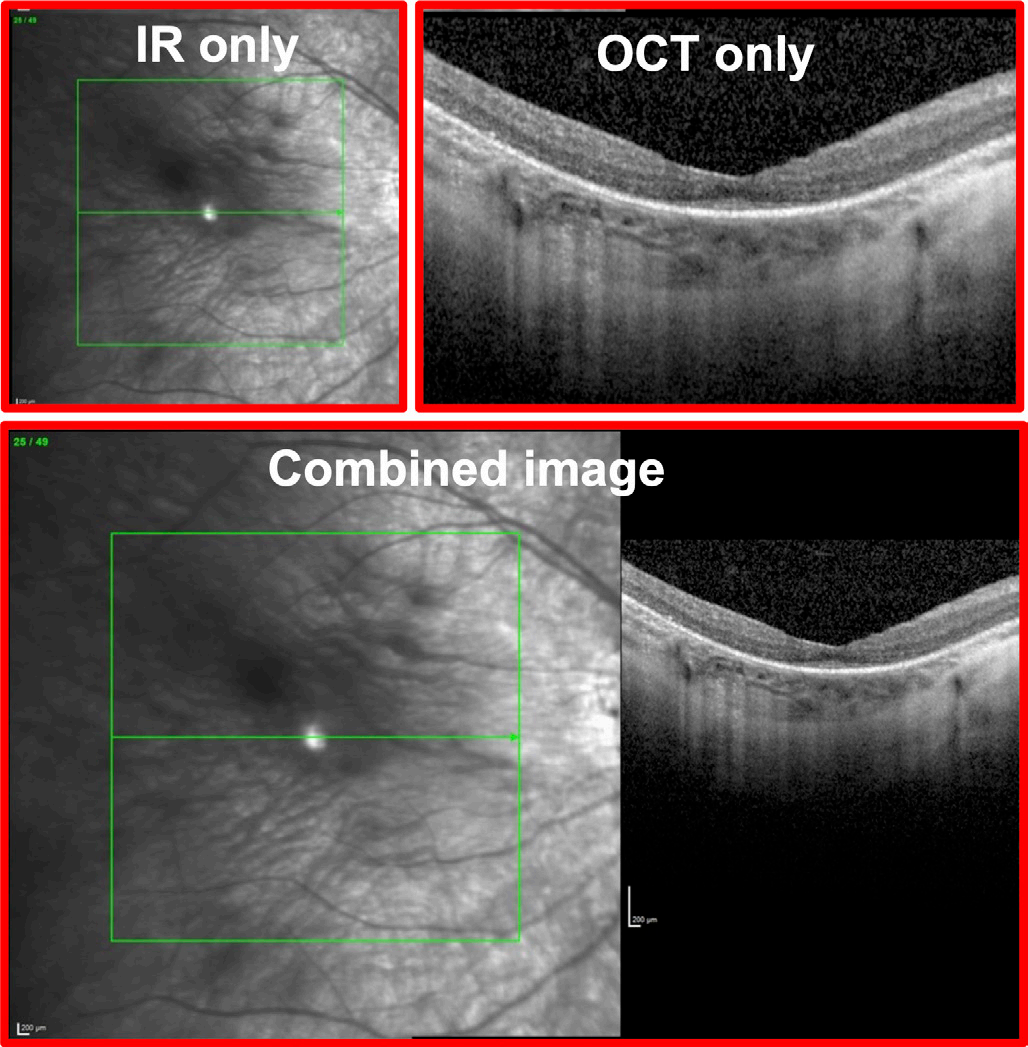
Sample images used as input data during neural network training and testing. The infrared (IR) only and optical coherence tomography (OCT) only images are shown in the top row. The combined image includes the IR and OCT images exported in a standardised combined format.

The corrected VA and cSLO imaging (Spectralis, Heidelberg Engineering, Heidelberg, Germany) data of each eye at each clinic visit was obtained via retrospective chart review. For each eye, both spectral domain OCT and en face IR imaging were obtained using the Heidelberg Spectralis machine. The foveal OCT line scan and the corresponding IR image of each eye at all available visits were exported in an uncompressed TIFF format in a deidentified fashion (1280×868 pixels and 24 bit/pixel). During image export using the commercial software that accompanied the Heidelberg Spectralis machine, the default export format was a combined image (CI), containing both the IR and OCT images, as shown in figure 1.

### Neural network training

A 10-way k-fold cross-validation was used to determine the optimal parameters for neural network training. Four pretrained neural networks were tested: AlexNet,^27^ DenseNet-161,^28^ ResNet-50, ResNet-152.^29^ The pretrained weights were based on the ImageNet training and read directly as part of the network loading in PyTorch. The number of epochs varied from 1 to 30. Three learning rates were tested, 0.01, 0.001 and 0.0001, a batch size of 16 was used. Stochastic gradient descent as the optimiser and cross-entropy loss as the loss function were used. The random seed was manually set for each of the Python packages to create a reliable comparison across runs. The area under the curve (AUC) of the receiving operator curve was measured over the 10-folds and reported as a mean and SD to determine the best set of parameters. This was repeated for the IR images only, OCT images only and CIs.

An optimal set of parameters was generated for each image type (IR, OCT and CI). Therefore, a separate network was trained for each image type (three separate networks in total) using the ResNet-152 (the best-performing neural network architecture during 10-fold cross-validation), a batch size of 16, learning rate of 0.001, stochastic gradient descent and a cross-entropy loss. The three trained networks (IR, OCT and CI) were used for further testing, both internal (JHU dataset) and external (AUMC dataset).

To evaluate the performance of the three networks, each network was tested twice, once against the held out portion of the JHU dataset (internal testing) and once against the entire AUMC dataset (external testing), for the binary classification task of distinguishing between Snellen VA 20/40 or better and worse than Snellen VA 20/40. The AUC was calculated along with the precision and recall. Gradient-based class activation maps were calculated^30^ during external testing to visually understand the spatial activation from the network.

All data processing and neural network training and prediction were accomplished in Python V.3.7 using PyTorch V.1.8 and related packages. The training was performed with a computer with dual GPU Tesla P100-PCIE with 12 GB RAM each.

## Results

This study included a total of 2918 images from 628 eyes from 314 patients. Of these, the training (JHU) database included 2569 images from 462 eyes from 231 patients (65% Caucasian; 23% Black; 6% Asian; 47% male). Within this cohort, the median age at the time of imaging was 52 years (range: 7–88 years) and the median Snellen VA was 20/40 (range: 20/16 to no light perception). Of the 2569 images, 62% were from eyes with Snellen VA 20/40 or better and 38% were from eyes with worse than Snellen VA 20/40. Within the JHU cohort, 197 patients had longitudinal OCT scans (median: 4) over a median follow-up period of 2.9 years.

The testing (AUMC) database included 349 images from 166 eyes from 83 patients (70% Dutch; 11% Middle Eastern; 6% African; 5% non-Dutch European; 4% Asian; 4% South American; 60% male). All 83 patients carried pathogenic mutations confirmed by genetic testing. The most commonly involved genes were: *USH2A* (19%), *RPGR* (13%), *CRB1* (8%), *RHO* (6%), *RP1* (6%), *EYS* (5%) and *MYO7A* (5%). Pathological mutations were found in two patients for each of the following genes: *PRPH2, SNRNP, PRPF31, ABHD12, RP2, SGSH, BBS1* and *NR2E3*. Pathological mutations were found in one patient for each of the following genes: *PDE6A, PDE6B, HGSNAT, AD4RV1, FDE6B, ABCA4, MERTK, RLBP1, PRPF31, CDH23, NPHP1, LRAT, RDH12, C80RF3T* and *FAM161A*. Within this cohort, the median age at the time of imaging was 38 years (range: 6–77 years; IQR 29–55 years) and the median Snellen VA was 20/32 (range: 20/16 to light perception; IQR 20/25 to 20/100). Of the 349 images, 52% were from eyes with Snellen VA 20/40 or better and 48% were from eyes with worse than Snellen VA 20/40. Within the AUMC cohort, 49 patients had longitudinal OCT scans (median: 2) over a median follow-up period of 1.2 years.

### Internal testing

After the 10-fold cross-validation experiments were completed, three versions of the network were trained (IR, OCT and CI). Optimal hyperparameters were first obtained during cross-validations on the splits of the training subset, and then the model was trained with all the data in the training subset. An internal testing, using a held-out JHU dataset, was performed. The AUC for distinguishing between Snellen VA 20/40 or better and worse than Snellen VA 20/40 was 0.83, 0.87 and 0.85 for IR, OCT and CI, respectively. The results of the internal testing are summarised in table 1.

**Table 1:** Internal test results on the Johns Hopkins University (JHU) dataset and external test results on the Amsterdam University Medical Centers (AUMC) dataset, using the ResNet-152 network

JHU				
**Modality**	**VA category**	**AUC**	**Precision**	**Recall**
Infrared only	Overall	0.83	0.78	0.77
	Snellen 20/40 or better		0.71	0.83
	Worse than Snellen 20/40		0.83	0.71
OCT only	Overall	0.87	0.76	0.76
	Snellen 20/40 or better		0.73	0.75
	Worse than Snellen 20/40		0.78	0.76
Combined	Overall	0.85	0.83	0.82
	Snellen 20/40 or better		0.76	0.89
	Worse than Snellen 20/40		0.89	0.76
**AUMC**				
**Modality**	**VA category**	**AUC**	**Precision**	**Recall**
Infrared only	Overall	0.78	0.69	0.63
	Snellen 20/40 or better		0.80	0.40
	Worse than Snellen 20/40		0.56	0.88
OCT only	Overall	0.87	0.79	0.79
	Snellen 20/40 or better		0.84	0.74
	Worse than Snellen 20/40		0.74	0.84
Combined	Overall	0.85	0.77	0.77
	Snellen 20/40 or better		0.78	0.78
	Worse than Snellen 20/40		0.75	0.75

Three versions of the network were trained and tested: infrared only, OCT only and combined image.

AUC, area under the curve; OCT, optical coherence tomography.; VA, visual acuity.

### External testing

**Figure 2:**
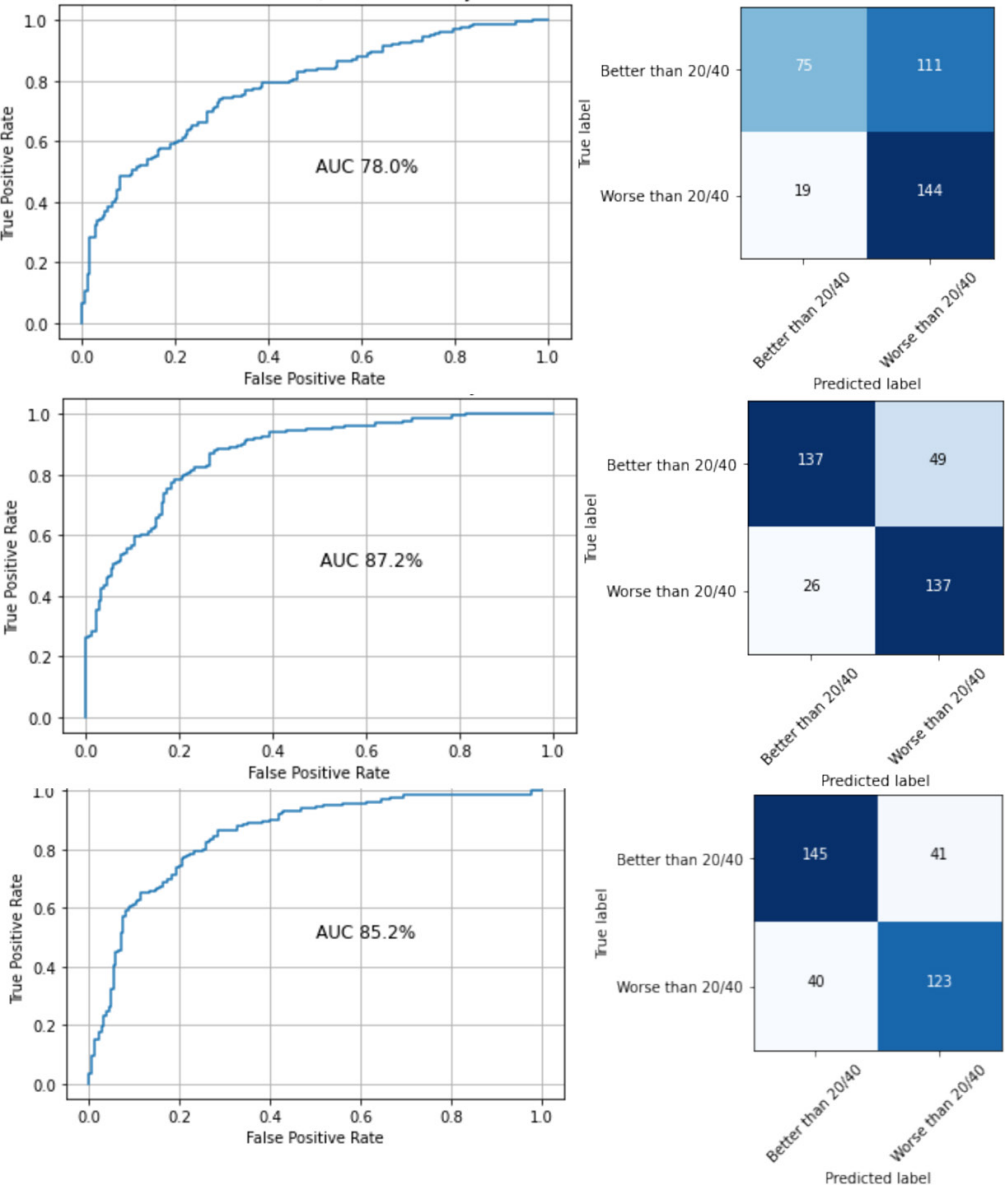
The receiver operating characteristic curves and corresponding confusion matrixes generated from external testing on the Amsterdam University Medical Centers (AUMC) dataset. Top (infrared); middle (optical coherence tomography); bottom (combined). AUC, area under the curve.

Using the same models that were used in internal testing, we tested our algorithms against the external dataset obtained from AUMC. The AUC for distinguishing between Snellen VA 20/40 or better and worse than Snellen VA 20/40 was 0.78, 0.87 and 0.85 for IR, OCT and CI, respectively. Of the 166 eyes in the test set, 96 eyes had serial images. The accuracy of our model for OCT images was 71% on an eye level. For an eye with serial images, it was counted as ‘correct’, only if all images generated from that eye were predicted correctly. The results of the external testing are summarised in table 1 and figure 2. Of the 349 images in the external test set, 52 contained structural abnormalities other than outer retinal atrophy: full-thickness macular hole (n=3), lamellar macular hole (n=11) and CME (n=38) that significantly distorted the foveal contour. Of these 52 images, 27 images (52%) received an incorrect prediction from the version of the network that involved only OCT images.

**Figure 3:**
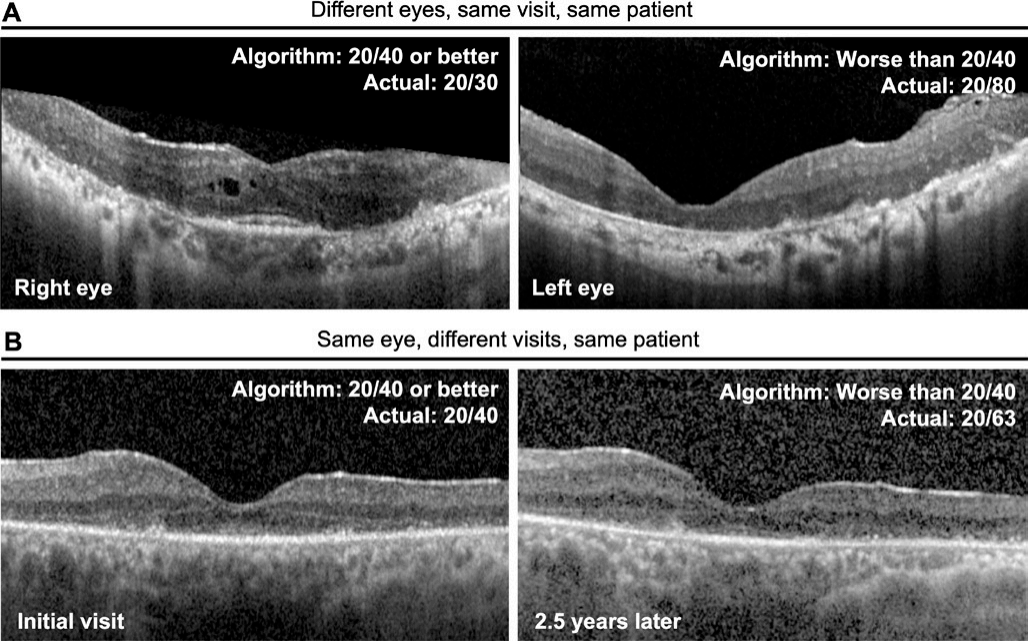
Examples of cases in which algorithm-predicted visual acuity (VA) correctly identified intereye or intervisit differences in actual VA. (A) Images obtained from the same subject at the same visit. Our algorithm correctly classified the right eye as ‘Snellen 20/40 or better’ (ground truth actual VA: 20/30) and the left eye as ‘worse than Snellen 20/40’ (ground truth actual VA: 20/80). (B) Images obtained from the same eye at visits that were 2.5 years apart. The actual VA decreased from Snellen 20/40 to 20/63 over this interval. Our algorithm correctly classified the VA at the initial visit as ‘Snellen 20/40 or better’ and the subsequent visit as ‘worse than Snellen 20/40’.

Herein, we present two examples of successful application of our algorithm. The first example (figure 3) shows the detection of a contemporaneous functional difference between the two eyes of a single patient. The patient had relatively asymmetric structural changes in the two eyes. The right eye (oculus dexter, OD) showed a residual EZ line in the fovea (Snellen VA 20/30). The left eye (oculus sinister, OS) showed a near complete loss of EZ line in the fovea (Snellen VA 20/80). Our algorithm correctly classified OD as Snellen VA 20/40 or better and OS as worse than Snellen VA 20/40. The second example in figure 3 shows the detection of a functional change over time in a single eye. The same eye was evaluated at two successive visits (2.5 years apart), over which the Snellen VA decreased from 20/40 to 20/63. Our algorithm correctly classified the earlier visit as VA 20/40 or better and the follow-up visit as worse than VA 20/40.

## Discussion

The data showed that a DL algorithm can be used to correlate structure with function using only cSLO OCT imaging data in patients with RP. Specifically, this algorithm was able to predict the presence or absence of visual impairment, based on the 20/40 cut-off that is defined by WHO and generally accepted in the USA and internationally. The algorithm appears to be able to detect contemporaneous interocular differences in VA, as well as temporal changes in VA. The ability of a DL algorithm to predict VA based on imaging has also been demonstrated in a recent study by Kawczynski *et al* 19 for neovascular AMD. However, the current study is, to our knowledge, the first demonstration of the application of DL to predict structure-function correlation in IRDs.

**Figure 4:**
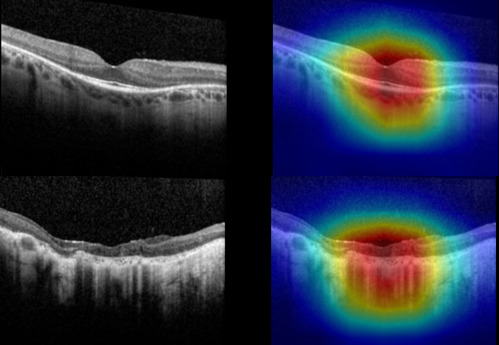
Sample Grad CAM visualisation of correct predictions during external testing with the Amsterdam University Medical Centers dataset. Top (Snellen 20/40 or better); bottom (worse than Snellen 20/40).

Our analysis showed that using CIs did not confer additional predictive power, as there was no improvement in AUC over using OCT images alone. Examination of the gradient-based class activation maps in the CIs showed strong activation on the OCT side in most images, suggesting that when our deep neural network was presented simultaneously with an IR and OCT image during training, it tended to ‘learn’ mostly from the OCT component. Examination of the gradient-based class activation maps in OCT-only images showed strong activation centred on the fovea and/or remaining EZ, suggesting that our model was learning from OCT features that were biologically meaningful and medically relevant. Sample visualisation of correct predictions are shown in figure 4. We have chosen Snellen VA 20/40 as the cut-off for the binary classification used in this study because this is a functionally meaningful cut-off. Snellen VA 20/40 is the cut-off for driver’s license requirements in many European countries and in most states in the USA.^31^ Vision worse than 20/40 has been shown to be a risk factor for limitations in instrumental ADL,^32^ and is often defined as visual impairment in population-based studies in the USA.^33 34^

While our study contributes novel information regarding the role of DL in functional prediction, other studies have looked at DL techniques in RP primarily focusing on disease detection, diagnosis and anatomic measurement. Examples include measurement of preserved photoreceptors on en face OCT,^35^ measurement of EZ width on OCT B-scans,^36^ diagnosis of RP using colour fundus photographs,^37 38^ differentiation of RP from Best vitelliform macular dystrophy, Stargardt disease and healthy controls on SW-FAF^39^ and to differentiate *EYS*-associated RP from *ABCA4*-associated and *RP1L1-*associated IRD based on OCT imaging.^40^ While quantitative measurement of traditional image-based clinical end points, such as the EZ width on OCT B-scans, is useful to track progression in RP, DL-based approaches may offer additional advantages such as pattern recognition of multiple features, including those that are difficult to quantify such as outer retinal hyper-reflective foci^41^ and signal hypertransmission due to outer retinal atrophy.

A DL algorithm that can accurately predict VA from OCT images could be valuable in terms of clinical care and clinical trials for RP. With further optimisation, DL-based OCT analysis could be potentially developed into an OCT ‘potential acuity metre’ and circumvent the impact of undercorrected refractive error, ocular surface disease and media opacity. DL-based OCT analysis may also provide data that could potentially support clinical management of patients with RP, such as estimating the potential VA improvement with cataract surgery. In the realm of clinical trials, the sensitivity and specificity of the DL algorithm can be adjusted to fit the needs of a particular research goal. For example, a high-specificity version of this algorithm can be used as a screening tool in clinical trial enrolment to ensure that only patients with a VA worse than 20/40 are included. Once a DL algorithm has been trained to accurately predict function from imaging, additional analyses can be done to identify novel imaging biomarkers that correlate with functional outcomes.

The strength of our study lies in the validation of our algorithm by a truly external independent dataset, which was meticulously curated and included only molecularly confirmed patients with RP with disease-causing variants. The main weakness of our study stems from its retrospective design, in which VA was measured in a clinical practice setting and thus lacked prospective standardisation and the age of disease onset was not recorded in most patients. At this stage of development, our algorithm can perform only binary classifications instead of multivalent classifications, although we picked a VA cut-off that is functionally meaningful. Also, the performance of our algorithm declined in the presence of additional structural abnormalities other than outer retinal atrophy such as significant CME.

These findings support the feasibility of developing a DL-based cSLO imaging metric that will correlate with VA in RP. In principle, this metric will incorporate multiple physiological and pathological anatomical structural features in RP eyes in an unbiased manner. Further research will clarify the utility of incorporating additional imaging modalities such as SW-FAF, and the ability of DL algorithms to predict other aspects of visual function that are clinically meaningful. If successfully developed, this DL metric could be taken as a unified clinically meaningful biomarker that is important in a practical sense to patients with RP, and therefore be favourably considered as a quantitative surrogate end point in observational or interventional clinical trials in RP.

With further optimisation, DL algorithms for structure-function correlation in RP and other ocular diseases may enhance the efficiency of recruitment efforts for clinical trials and support clinical decision-making regarding management strategies for visual impairment. The ability of this algorithm to detect a functional difference between eyes, and over time in the same eye, suggest a possible future role in long-term patient monitoring and evaluating treated versus control eyes in clinical trials. As the next step, we plan to include fundus autofluorescence images, more training OCT images with CME, increase the size of the training dataset to allow for training of the neural network in regression mode to predict the exact VA on a numeric scale and to train a model that can predict other important visual functions such as the size of the remaining visual field.

## Conclusions

A DL algorithm can discriminate between two levels of VA with relatively high sensitivity and specificity, using only a single-slice transfoveal OCT image as the input data. Specifically, the DL algorithm was able to detect visual impairment based on a VA cut-off of 20/40. The role of multimodal imaging input in improving algorithm performance is unclear at present. These data establish the feasibility of predicting structure-function correlation based on OCT images in patients with RP.

## Data Availability

All data relevant to the study are included in the article or uploaded as supplementary information.
